# Clear Cell Carcinoma Arising from Ovarian and Thoracic Endometriosis: A Case Report and Review of Literature

**DOI:** 10.1155/2022/7624305

**Published:** 2022-06-29

**Authors:** Samantha Mendoza Stanteen, Taemee Pak, Hao Chen, Matthew Carlson, Jessica Lee

**Affiliations:** ^1^University of Texas Southwestern Medical School, 5323 Harry Hines Boulevard, Dallas, TX 75390, USA; ^2^University of Texas Southwestern Medical Center, Department of Radiology, 5323 Harry Hines Boulevard, Dallas, TX 75390, USA; ^3^University of Texas Southwestern Medical Center, Department of Pathology, 5323 Harry Hines Boulevard, Dallas, TX 75390, USA; ^4^University of Texas Southwestern Medical Center, Department of Obstetrics and Gynecology, Division of Gynecologic Oncology, 5323 Harry Hines Boulevard, Dallas, TX 75390, USA

## Abstract

We report a case of stage IVB ovarian clear cell carcinoma in a 35-year-old female with a long-standing history of biopsy-proven pelvic and thoracic endometriosis. At the time of her ovarian cancer diagnosis, her tumors were found to be isolated to the sites of her previously known endometriotic lesions, suggesting that malignant transformation of her endometriosis to ovarian cancer had occurred. She underwent primary tumor debulking, then received six cycles of intravenous carboplatin and paclitaxel, and is now free of disease. We have conducted a literature review of ovarian cancers arising from endometriosis as well as a summary of the molecular basis on the relationship between endometriosis and malignant ovarian carcinoma.

## 1. Introduction

In 2022, it is estimated that there will be 19,880 new cases of ovarian cancer diagnosed in the US, and despite advances of treatment, an estimated 12,810 women will die of this disease [1]. Ovarian clear cell carcinomas (CCC) account for 5-10% of epithelial ovarian cancers and are at times thought to arise from malignant transformation of a benign precursor lesion such as endometriosis. Endometriosis, a condition affecting approximately ten percent of reproductive aged women [2, 3], is defined by the presence of benign endometrial glands and stroma outside of the uterus. It is most often found in the pelvic cavity; however, documented cases of endometriosis outside of this region blur the limits of the condition. The thoracic cavity is a known extrapelvic site of endometriosis, and catamenial pneumothorax is the most common clinical presentation of thoracic endometriosis [4–6]. While most epithelial ovarian cancers spread primarily via exfoliation to the peritoneal cavity and through lymphatic channels [2], the pattern of spread of endometriosis is less predictable, and the mechanism is less understood. There are currently several theories proposing retrograde menstruation, metaplastic changes, and/or hematogenous and lymphatic dissemination as mechanisms of endometriosis spread to extrapelvic structures [2]. We report a case of a patient with ovarian and thoracic endometriosis that transformed into clear cell adenocarcinoma, limited to the sites of her known endometriotic lesions, with no other sites of disease found in the abdomen or retroperitoneum.

## 2. Case

**Figure 1:**
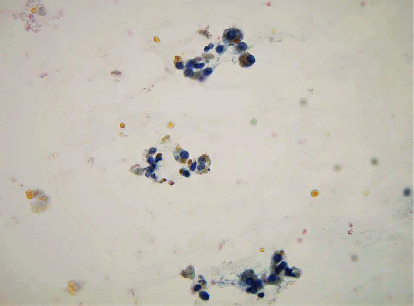
Hemosiderin laden macrophages in pleural fluid consistent with endometriotic cells.

A 35-year-old woman, gravida 0, with a long history of dysmenorrhea and chronic pelvic pain presented to the emergency room (ER) with shortness of breath. A transvaginal ultrasound revealed bilateral adnexal masses consistent with endometriomas. CT angiography of the chest showed a large right pleural effusion with compressive atelectasis and no evidence of pulmonary emboli. The patient's history and ultrasound findings raised concern for thoracic endometriosis as the etiology of the pleural effusion. A subsequent thoracotomy revealed a hemorrhagic effusion as well as chocolate-brown diaphragmatic pleural implants, which were excised. Pathologic examination of these implants was consistent with pleural involvement of endometriosis based on the presence of hemosiderin-laden macrophages (Figure 1).

Over the following six years, the patient received eight courses of a gonadotrophin-releasing hormone (GnRH) agonist to treat her endometriosis while maintaining future fertility. She was then lost to follow-up. Two years later, she represented with a recurrent pleural effusion and was restarted on the GnRH agonist. She received twelve doses in total.

**Figure 2:**
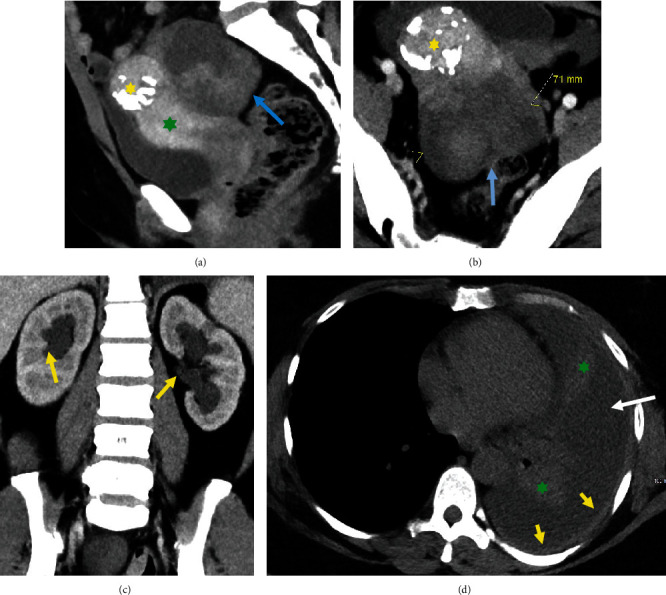
Sagittal (a) and axial (b) contrast-enhanced CT images of the pelvis demonstrate a large heterogeneous lobulated mass (blue arrow) posterior to the uterus (green asterisk). The uterus contains a coarsely calcified exophytic fibroid (yellow asterisk). (c) Bilateral mild-moderate hydroureteronephrosis with ureteral collapse at the level of the pelvic mass. (d) Axial unenhanced CT image of the chest demonstrates a large left pleural effusion (white arrow) with compressive atelectasis of the lingula and left lower lobe (green asterisk) as well as left posterior pleural thickening (yellow arrow).

Nine years following her initial presentation, the patient was seen in the ER with severe flank pain. On physical examination, the patient was found to have diffuse abdominal tenderness with mild distention. She was unable to tolerate a pelvic examination due to pain. On laboratory testing, her serum creatinine was notably elevated up to 2.4 mg/dL, and her serum CA-125 was within normal limits at 28.6 units/mL. CT studies of the abdomen and pelvis showed an enlarged complex pelvic mass (Figures 2(a) and 2(b)) causing bilateral hydroureteronephrosis (Figure 2(c)) as well as omental stranding and ascites, concerning for carcinomatosis. The CT of the chest demonstrated unchanged pulmonary nodules as well as a recurrent left pleural effusion and left pleural thickening (Figure 2(d)).

The patient subsequently underwent an exploratory laparotomy, total abdominal hysterectomy, bilateral salpingo-oophorectomy, omentectomy, pelvic and para-aortic lymph node dissection, and tumor debulking to no gross residual disease. Intraoperatively, bilateral ovaries masses with dilated fallopian tubes were encountered as well as dense adhesions involving the ovaries and retroperitoneal fibrosis. The uterus was fixed in the cul-de-sac and adherent to the bladder, bilateral ureters, sigmoid colon, and bilateral ovaries. Contrary to her CT findings, the patient's omentum was free of disease, and there was no gross disease outside of the pelvis.

**Figure 3:**
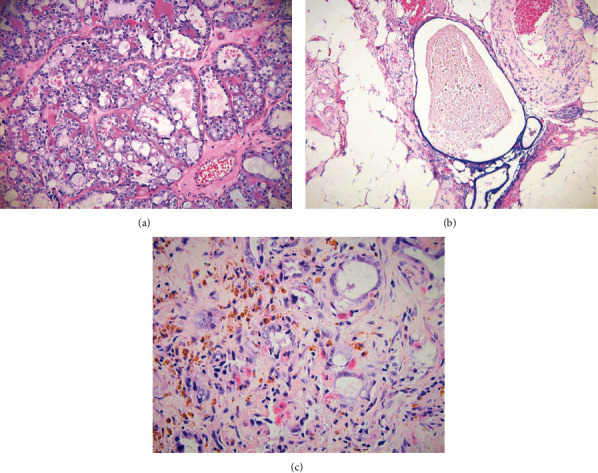
(a) The tumor in the ovary consists of glands lined with low cuboidal, polyhedral, hobnail or flattened cells with uniform nuclear atypia, and variable mitotic activity. Cytoplasmic clearing of tumor cells and background hyalinized stroma is prominent. (b) Extensive endometrioses were noted in multiple anatomical locations. The omentum is pictured here. (c) The tumor in the pleura consists of glands lined with tumor cells similar to those of ovarian cancer. Background hemosiderin-laden macrophage is prominent in this biopsy.

Final pathology revealed clear cell adenocarcinoma involving both ovaries with capsular involvement (Figure 3(a)). On gross examination, the left ovary measured 10.4 × 9.1 × 2.3 cm and the right ovary measured 10.0 × 9.2 × 3.9 cm. Both ovaries were disrupted with extensive tumor surface involvement. The tumor appeared to completely obliterate both ovaries and fallopian tubes. There was also extensive endometriosis and surface adhesions involving the periadnexal soft tissue, which also revealed endometriosis. The tumor demonstrated a typical clear cell carcinoma immune profile (Napsin-A: strong diffusely positive; racemase: strong diffusely positive; estrogen receptor: negative; progesterone receptor: negative). All other biopsies and lymph nodes were negative for carcinoma but did reveal extensive endometriosis (Figure 3(b)).

Postoperatively, the patient was noted to have a persistent supplemental oxygen requirement due to an enlarging pleural effusion. Her effusion remained despite the placement of a pigtail pleural catheter. Due to the concern for a malignant pleural effusion causing an entrapped lung, the patient underwent a video-assisted thoracic exploration with biopsies and placement of a tunneled pleural catheter. She was found intraoperatively to have multiple pleural nodules, which on final pathology confirmed metastatic clear cell carcinoma (Figure 3(c)). Based on her pathology results, a diagnosis of stage IVB clear cell ovarian carcinoma was rendered.

The patient received six cycles of intravenous carboplatin and paclitaxel, and CT imaging following her chemotherapy regimen was negative for residual or recurrent malignancy. The pleural catheter was removed after her third cycle of chemotherapy after the resolution of her pleural effusion. The patient underwent germline genetic testing, which was found to be negative for pathogenic variants. Additional somatic tumor testing on the tissue obtained during her tumor debulking surgery was negative for BRCA1 and BRCA2 and demonstrated homologous recombination proficiency. The tumor testing did reveal mutations in ARID1A and PIK3CA. She currently remains without evidence of disease for 18 months since the completion of her adjuvant chemotherapy.

## 3. Discussion

Epithelial ovarian cancers metastasize primarily by exfoliation within the peritoneal cavity and also by lymphatic spread. As such, surfaces of intraperitoneal structures as well as retroperitoneal lymph nodes are the most frequent locations of ovarian cancer metastases [2]. In this case, the peritoneal cavity and retroperitoneum were spared despite the presence of metastases in the thoracic cavity. This presentation suggests that the clear cell carcinomas (CCC) in this patient arose directly from the endometriotic implants and supports the idea that CCC arising from endometriosis behaves differently from other epithelial ovarian tumors. Much remains unknown about endometriosis and endometriosis-associated CCCs to explain the transformation from benign endometriosis to invasive cancer.

Endometrioid and clear cell carcinomas are the histologic subtypes of ovarian cancer that can arise from endometriosis, and they are collectively referred to as endometriosis-associated ovarian cancers (EAOC) [3]. EAOCs, as well as mucinous and low-grade serous carcinomas, are considered to be type I ovarian tumors which are characterized as slow-growing, confined neoplasms derived from a precursor “borderline” lesion. Type 1 tumors are associated with mutations in KRAS, ARID1A, PIK3CA, PTEN, and BRAF; whereas the majority of type 2 tumors are associated with mutations in TP53 [7].

Anglesio et al. conducted molecular studies of seven cases with synchronous endometriosis and clear cell or endometrioid carcinoma which shared identical somatic mutations (15-98%) between malignant and endometriotic regions. They concluded that a clear ancestral relationship existed between at least one endometriotic implant and the cancer it developed into [8]. Kato et al. examined HNF-1beta expression in ovarian tumors and found it to be a specific marker for clear cell ovarian tumors regardless of the clinical stage, and expression of HNF-1beta was also observed in atypical or reactive endometriosis. They proposed that early transformation of endometrial tissue to a clear cell lineage and expansion of these lesions may be the basis for the transformation of CCC from endometriosis and explain why some endometriotic lesions develop into CCC while others develop into endometrioid carcinomas [9]. EAOC have also been shown to have a high frequency of ARID1A gene mutations, with CCCs having a higher frequency than endometrioid. Atypical endometriotic implants adjacent to HNF-1beta positive CCC lesions have also been found to carry ARID1A mutations despite being negative for HNF-1beta expression. This suggests that a mutation in the ARID1A gene may be an early change that occurs in the progression from endometriosis to cancer [10]. In our case, molecular studies conducted on the excised malignant lesion revealed an ARID1A mutation without loss of heterozygosity (LOH), which is more often associated with type II tumors such as high grade serous carcinomas [10]. While the exact mechanism of transformation remains to be elucidated, these studies suggest that somatic genetic changes may play a pivotal role in the progression of endometriosis to an endometrioid or CCC.

It appears that our patient's endometriomas as well as the endometriotic implants in her pleural space transformed into invasive CCC. She initially had declined definitive treatment of her endometriosis due to her desire to maintain future fertility. For some premenopausal female patients, the prospect of losing the ability to have children can represent a significant psychological and emotional burden especially when coupled with a new cancer diagnosis. Fortunately, there have been many advancements in fertility preservation including oocyte and embryo cryopreservation that can be performed swiftly without delaying the initiation of cancer treatment. Ovarian tissue cryopreservation is a rapidly developing technology that will likely evolve to become standard therapy in the near future [11]. A multidisciplinary team including reproductive endocrinology, gynecology, oncology, surgery, and psychology is recommended in the care of these women [11–13].

Thoracic endometriosis is a rare form of extrapelvic endometriosis and is found concurrently with pelvic disease in only 30-50% of cases of thoracic endometriosis [14]. As in this case, catamenial pneumothorax is the most common clinical presentation of thoracic endometriosis followed by catamenial hemothorax, catamenial hemoptysis, and lung nodules. First-line therapy of thoracic disease is the same as the therapy for pelvic endometriosis via hormonal suppression with progestins, oral contraceptives, GnRH agonists, or newer GnRH antagonists. Refractory cases are treated surgically through video-assisted thoracoscopic surgery (VATS) or open surgery to resect removal of endometriotic tissue.

The time course from the presence of benign endometriosis to CCC in our patient suggests that the transformation may have occurred simultaneously in the chest and ovaries. In addition, the presence of CCC limited to the locations of her prior endometriotic implants without infiltration in areas where there had not been endometriosis present previously further supports that CCC arising from endometriosis behaves differently than other epithelial ovarian tumors. Endometriotic lesions being precursors for EAOCs, and the postulation that these lesions carry prooncogenic mutations, have an important implication for the treatment of CCC arising from endometriosis. It can then be concluded that when one endometriotic implant is found to be malignant, removal of all endometriotic implants may be necessary to prevent future development of clear cell cancer at other sites. Foregoing systemic lymphadenectomy may be considered especially if this may lead to increased perioperative morbidity.

## 4. Conclusion

While the malignant transformation of endometriosis is relatively rare (occurring in approximately 1% of cases), the prevalence of endometriosis in reproductive aged women warrants a better understanding of the carcinogenesis of endometriotic lesions [15]. Unfortunately, in our case, no tumor genomics were performed on the endometriotic tissue prior to the development of CCC, and therefore no comparison could be made between the mutations present in malignant and benign endometriotic tissue. Performing these tests in future similar presentations may contribute to our understanding of specific genetic alterations. Further investigation into somatic genomics as well as the role of the microenvironment is necessary in order to identify the women who are at risk of having their endometriosis transform into malignant invasive cancer.

## Data Availability

The authors declare that data supporting the findings of this study are available upon request.
